# Waterborne Diseases in a Rural Community in Pakistan: Awareness, Impact, and Corrective Measures

**DOI:** 10.7759/cureus.93154

**Published:** 2025-09-24

**Authors:** Rashk E Hinna, Rao Saad Ali Khan, Maham Javed, Zoya Ali Khan, Uzair Ali Khan

**Affiliations:** 1 Gastroenterology, Rahbar Medical and Dental College, Lahore, PAK; 2 Gastroenterology, Pak Emirates Military Hospital, Rawalpindi, PAK; 3 Internal Medicine, Rahbar Medical and Dental College, Lahore, PAK; 4 Science, Lahore Grammar School, Lahore, PAK; 5 Science, Carleton College, Northfield, USA

**Keywords:** community medicine & public health, diarrhea, drinking water source, rural area, waterborne infections

## Abstract

Background: This study aimed to assess the occurrence of waterborne illnesses in the rural community along the Hudiara Drain in Lahore, analyze the relationship between drinking water sources and waterborne illnesses, and evaluate the community's readiness to pay for improved water and sanitation facilities.

Method: A cross-sectional study was conducted from August 2023 to July 2024 in the area along Hudiara Drain (2 km off Bark Road on the left), Lahore, Pakistan. Primary data were collected through a structured questionnaire administered to 102 randomly selected households from a total of 450. The questionnaire covered socioeconomic demographics, water sources, waterborne disease prevalence, awareness of water quality, and willingness to pay for improved water and sanitation facilities. Statistical analysis was performed using IBM SPSS Statistics for Windows, Version 27 (Released 2020; IBM Corp., Armonk, New York) to generate descriptive statistics and graphical representations of key findings.

Results: The majority of respondents (89.2%) were male, with a mean age of 44.6 ± 11.8 years. The primary drinking water source was hand pumps (47%), followed by a combination of tube wells and hand pumps (32.4%). A total of 75 (62.5%) cases of waterborne infections were reported, with diarrhea being the most prevalent (36.3%), followed by both diarrhea and dysentery (9.8%), typhoid fever (6.9%), hepatitis A (2%), and hepatitis E (2%) infections. A strong association was observed between drinking water sources and disease prevalence, with hand pump users experiencing the highest number of infections. Despite widespread awareness of poor water quality (56.9%), corrective measures such as boiling water were not commonly practiced (25.5%). Notably, 79.4% of respondents were unwilling to pay for a pipeline water supply and sewerage system, citing financial constraints and reliance on government intervention.

Conclusion: The findings highlight the major burden of waterborne diseases in the community, with poor water quality and inadequate sanitation systems contributing to high infection rates. While awareness of water safety issues is prevalent, financial limitations and government dependence hinder corrective actions.

## Introduction

Waterborne infectious diseases primarily affect the human digestive system and are transmitted through contaminated water, water-related foods, and fruits and vegetables [1]. These diseases remain among the leading causes of mortality worldwide [2]. Approximately 780 million people worldwide lack access to safe drinking water, while 1.4 million lives are lost annually due to diseases associated with inadequate water, sanitation, and hygiene in households, healthcare facilities, and schools [3]. Additionally, around 2.5 billion individuals in developing countries lack proper sanitation [2,4].

Pakistan, endowed with abundant surface and groundwater resources, relies heavily on water for growth and development across various sectors. However, advancements in technology, along with the coexistence of sanitation and drainage systems, have contributed to the presence of various impurities in drinking water, including physical, chemical, and biological contaminants. Among these, biological impurities pose the greatest threat, as they can lead to severe health issues and even fatalities [5]. Bacterial pathogens, such as *E. coli*, *Salmonella*, *Shigella*, *Cryptosporidium*, and *Campylobacter*, are the primary causes of waterborne diseases, including diarrhea, hepatitis, cholera, typhoid, malaria, salmonellosis, dysentery, schistosomiasis, and giardiasis, all of which are increasingly prevalent [6]. It is estimated that approximately 30% of all diseases and 40% of deaths in the country are attributed to poor water quality. Diarrhea, a major waterborne disease, remains the leading cause of mortality among infants and children, while one in every five citizens experiences health issues linked to contaminated water [6]. Furthermore, diarrhea kills approximately two million people per year, accounting for 4% of global mortality [7].

In rural areas, the absence of proper water supply and sewerage systems makes water contamination a persistent issue. Factors such as infiltration, leaching, surface runoff, and leakage from inadequate sewage disposal systems contribute significantly to groundwater pollution [8]. Poor water quality has been linked to frequent disease outbreaks across many developing regions, including Pakistan, necessitating urgent interventions. Establishing drinking water systems that meet water quality standards can significantly reduce the prevalence of waterborne diseases [9]. Studies have shown that effective water and sanitation management practices can lower the incidence of diarrhea by one-third to one-fourth [10]. However, the success of these interventions depends on community participation and policy adoption. People's willingness to pay for improved water and sanitation services serves as an indicator of how much they value these resources. Understanding the relationship between willingness to pay and water quality can help service providers address pressing challenges and plan for both immediate and long-term improvements [9].

The general objective of this study is to examine the prevalence of waterborne diseases in the study area. Specifically, the study aims to assess the relationship between different drinking water sources and waterborne illnesses. In addition, it seeks to evaluate the community's willingness to pay for a pipeline water supply and sewerage system for disease prevention.

## Materials and methods

Study area and duration

**Figure 1:**
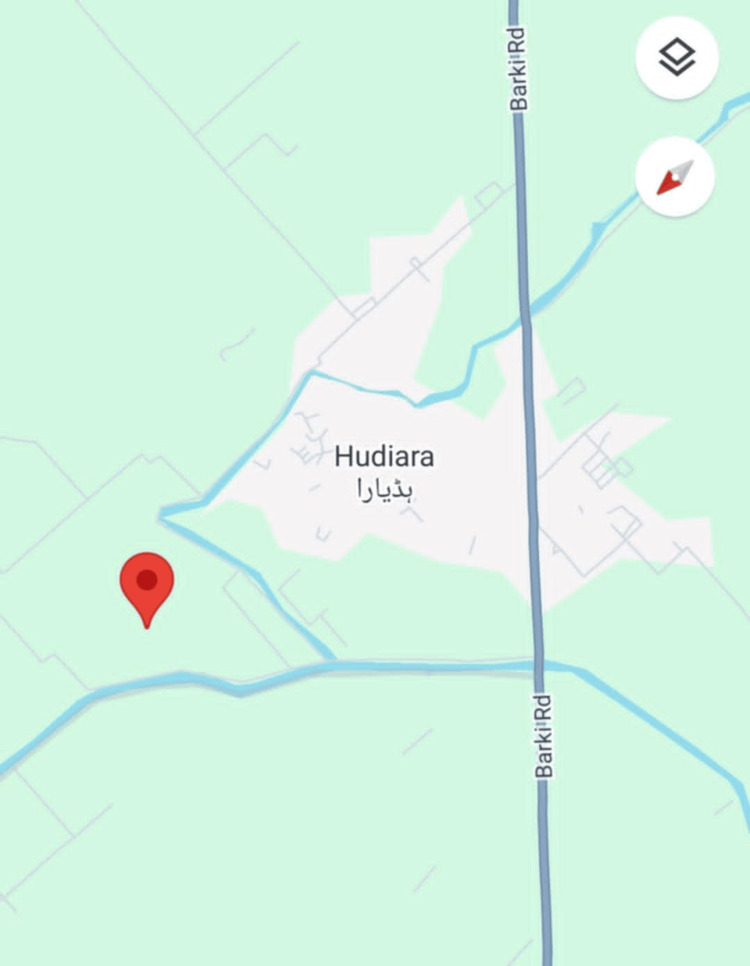
Hudiara Drain - study area Source: Google Maps (https://www.google.com/maps), 2025

This study was conducted in a rural community located along Hudiara Drain (2 km off Bark Road, on the left), Lahore, with geographical coordinates of 31°28'36"N and 74°30'45"E, from August 2023 to July 2024 (Figure 1). This community faces a lack of basic amenities, including a public water supply, proper sanitation, and a functional sewerage system, along with various socioeconomic challenges.

Study design and data collection

The study utilized primary data sources, employing a structured questionnaire to evaluate awareness, impact, and corrective measures related to waterborne diseases. The questionnaire covered respondents' demographics, including age, gender, and monthly income, as well as information on sources of drinking water, perceptions of water quality, and the types of waterborne infections experienced at the time of the survey. To ensure clarity and consistency, waterborne illnesses were explained to respondents in simple terms using common names and basic symptom descriptions (e.g., diarrhea as frequent loose stools, dysentery as the presence of blood in the stool). Respondents were also provided with a short list of common waterborne conditions, such as typhoid, cholera, and gastroenteritis, to standardize reporting. The questionnaire was developed based on a review of relevant literature and previously used tools, then pilot-tested on 10 households from a nearby community (excluded from the final study) to assess clarity and cultural appropriateness. Necessary refinements were made before final administration. Willingness to pay (WTP) for a pipeline water supply and sewerage system was assessed through a direct close-ended question.

The sample size was calculated using a standard single population proportion formula, assuming a 50% expected prevalence of waterborne diseases, a 95% confidence level, and a 10% margin of error, which resulted in a minimum required sample size of 97 households. To account for possible non-responses, 102 households were finally included. Households were selected through simple random sampling: a complete list of 450 households in the community was obtained, unique identifiers were assigned, and random numbers were generated to select the study sample. The respondents were primarily the heads of households.

Ethical consideration

The anonymity of respondents was guaranteed, and their consent was secured through customized consent forms.

Statistical analysis

All the data obtained through the questionnaire were analyzed using IBM SPSS Statistics for Windows, Version 27 (Released 2020; IBM Corp., Armonk, New York), to demonstrate graphical representations of various data characteristics, frequencies, percentages, and descriptive analysis.

## Results

Among 102 respondents, there were 89.2% males, with a mean age of 44.6 ± 11.8 years. Table 1 presents the detailed socioeconomic demographics.

**Table 1:** Socioeconomic variables of respondents

Variables	Category	Frequency (n)	Percentage (%)
Age (years)	Mean ± SD	44.6 ± 11.8	
Age group (years)	21–35	24	23.5
	36–50	48	47.1
	51–65	23	22.5
	66–80	7	6.9
Gender	Male	91	89.2
	Female	11	10.8
Education	Uneducated	69	67.6
	Primary	18	17.6
	Middle	10	9.8
	Matric	5	4.9
Total income (US dollars)	36–72	6	5.9
	73–108	41	40.2
	>108	55	53.9

**Figure 2:**
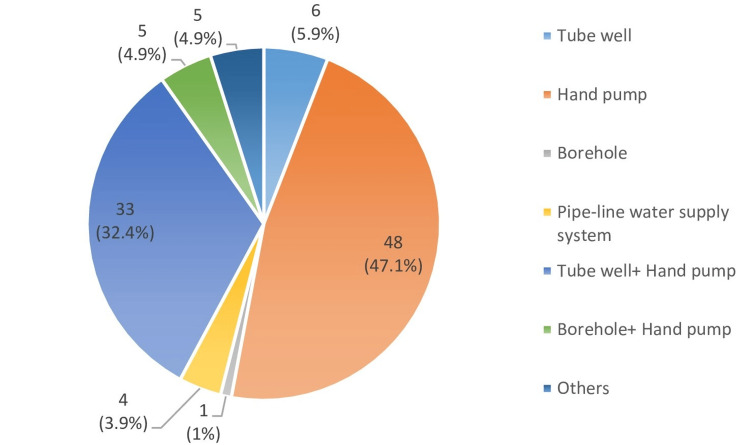
Sources of drinking water

Figure 2 illustrates the diverse drinking water sources used by the community, with hand pumps being the most prevalent, used by 47% of the population, followed by a combined use of tube wells and hand pumps (32.4%), while only 1% of the population relies on boreholes.

**Figure 3:**
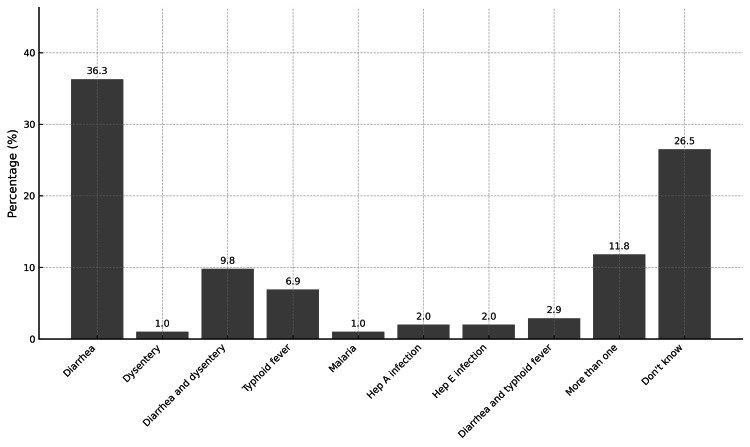
Waterborne diseases

Figure 3 presents the distribution of waterborne diseases in the community, with diarrhea emerging as the most prevalent (36.3%). A smaller proportion (9.8%) experienced both diarrhea and dysentery, while typhoid fever affected 6.9% of respondents. Meanwhile, 11.8% reported more than one waterborne disease, and 26.5% were unsure about their specific condition, highlighting the burden of waterborne illnesses and the need for improved water quality and sanitation measures in the study area.

**Figure 4:**
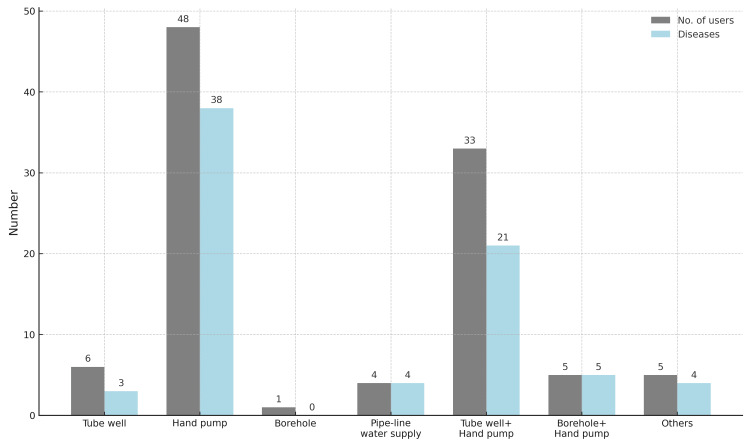
Comparison of drinking water sources and associated waterborne illness

Figure 4 depicts a strong association between drinking water sources and the prevalence of diseases. Hand pump users reported the highest number of infections (38 cases), with diarrhea being the most common ailment. Those relying on a combination of tube wells and hand pumps also showed a significant number of infections (21 cases), with typhoid fever and diarrhea being frequently reported. However, individuals using borehole water did not report any infections, indicating a potential difference in contamination levels.

The distribution of waterborne infections among users based on the source of their drinking water is presented in Table 2. A total of 75 (62.5%) cases of waterborne infections were recorded among the surveyed population. The most frequently reported infection was diarrhea (36.3%), followed by diarrhea and dysentery (9.8%).

**Table 2:** Distribution of waterborne infections by water source among users

Sources of Drinking Water	Total Users, n	Types of Waterborne Infections					
		Diarrhea, n (%)	Dysentery, n (%)	Typhoid, n (%)	Hepatitis A/E, n (%)	Multiple infections, n (%)	Uncertain, n (%)
Tube well	6	2 (33.3)	0	1 (16.7)	0	0	3 (50)
Hand pump	48	21 (43.8)	1 (2.1)	1 (2.1)	3 (6.3)	12 (25)	10 (20.8)
Borehole	1	0	0	0	0	0	1 (100)
Tube well + Hand pump	33	8 (24.2)	0	4 (12.1)	0	8 (24.2)	12 (36.4)
Borehole + Hand pump	5	2 (40)	0	1 (20)	1 (20)	1 (20)	0
Pipeline water supply system	4	2 (50)	0	0	0	2 (50)	0
Others	5	2 (40)	0	0	0	2 (40)	1 (20)

Table 3 highlights the community's awareness of water quality and the corrective measures taken to ensure the provision of safe drinking water. A majority (56.9%) perceived their drinking water as poor and not free from pathogens, while 37.3% were unsure about its safety. The need for a proper pipeline water supply and sewerage system was widely recognized, with 71.6% expressing the necessity for it. Despite concerns over water quality, corrective measures were not commonly practiced. Only 15.7% of individuals reported taking precautions to ensure the safety of their drinking water. Interestingly, despite acknowledging poor water quality, 79.4% of respondents were unwilling to pay for a pipeline water supply and sewerage system. The findings indicate that, while there is widespread awareness of poor water quality and sanitation issues, financial constraints and a reliance on government intervention hinder the implementation of effective corrective measures and infrastructure improvements (Table 3).

**Table 3:** Awareness and corrective measures by community

Category	Variables	Response	N (%)
Awareness	Perception of water quality	Good (Free of pathogens)	6 (5.9)
		Poor (Not free of pathogens)	58 (56.9)
		Not sure	38 (37.3)
	Conditions of sewerage disposal system in the area	Poor	57 (55.9)
		Good	15 (14.7)
		Not sure	30 (29.4)
	Perceived need for a pipeline water supply and sewerage system	Yes	73 (71.6)
		No	4 (3.9)
		Not sure	25 (24.5)
Corrective measures	Drinking water safety measures	Yes	16 (15.7)
		No	40 (39.2)
		Not sure	46 (45.1)
	Water boiled before drinking	Yes	26 (25.5)
		No	76 (74.5)
	Water sample taken for analysis	Yes	37 (36.3)
		Not sure	65 (63.7)
	Willingness to pay for a pipeline water supply and sewerage system	Not willing	81 (79.4)
		Not sure	21 (20.6)
	Barriers to improvement	Cannot afford the cost	40 (39.2)
		Believe it is the government’s responsibility	15 (14.7)
		Both of the above reasons	25 (24.5)
		Uncertain about its necessity	22 (21.6)

## Discussion

Water is an essential resource for human survival. However, increasing water insecurity is negatively impacting health standards and making survival more difficult. The lack of access to clean water, proper sanitation, and hygiene services is becoming more severe, particularly in rural areas and informal urban settlements worldwide, including Pakistan [11]. Contaminated water plays a significant role in the spread of waterborne diseases, including diarrhea, cholera, dysentery, hepatitis A, polio, and typhoid, exacerbating the global disease burden [12]. In 2019, approximately 884 million people lacked access to clean drinking water, exacerbating the risk of waterborne illnesses. The poor quality of drinking water directly increases the likelihood of contracting such diseases [13]. Annually, approximately 1.8 million individuals succumb to cholera and diarrhea, while 3,900 children lose their lives each day due to inadequate water and sanitation conditions [14].

The distribution of safe drinking water in Pakistan is highly uneven, with only 20% of the population having access to clean and quality water. Consequently, the majority of the population is left with substandard water sources to meet their daily needs, undoubtedly exposing them to various diseases and other toxic effects of contaminated water, which are exacerbated by anthropogenic activities [15]. Despite inadequate drinking water and sanitation facilities, the lack of medical services remains a significant concern in rural areas of Pakistan, which is responsible for the outbreak of diseases.

In this study, 47% of respondents relied on a single water source, i.e., a hand pump, while 32.4% used a combination of tube wells and hand pumps, and only 1% depended on boreholes. Alarmingly, despite a sample size of 102 households, 62.5% of individuals had experienced waterborne diseases, a finding consistent with the research of Shahzad and his colleagues, who reported a 60% prevalence of such diseases in their study population [16]. Khan et al. highlighted that many communities depend on outdated water supply schemes, with 73% using well water, 13% relying on hand pumps, 11% using spring water, and 3% sourcing water from rivers or streams. This aging infrastructure has led to widespread waterborne diseases such as hepatitis, intestinal infections, diarrhea, dysentery, cholera, typhoid fever, jaundice, and various skin conditions, affecting children the most, followed by younger and older adults [17].

The high contamination levels in hand pump water sources can be attributed to several factors. Firstly, many hand pumps in rural areas are installed at shallow depths, making them highly susceptible to contamination from nearby sewage systems, agricultural runoff, and industrial waste. Secondly, poor maintenance and a lack of proper sealing allow surface contaminants, including bacteria and heavy metals, to infiltrate the groundwater. Additionally, human activities such as open defecation, improper waste disposal, and the use of fertilizers and pesticides in nearby fields contribute to the presence of coliform bacteria and other harmful pathogens in the water [15]. The close proximity of hand pumps to latrines and animal waste further exacerbates the issue, leading to an increased prevalence of waterborne infections among users. Unlike borehole water, which is often extracted from deeper and relatively safer underground sources, hand pumps frequently draw from shallow, contaminated aquifers [18].

A study carried out by Shah et al. documented that 34% of patients presented with diarrhea, 25% with cholera, 19% with dysentery, 14% with typhoid, and 8% with hepatitis. Children were the most affected demographic due to unsafe drinking water [19]. Similarly, Butt and Khair found that around 75% of households in Quetta City experienced waterborne diseases, with 32% of households reporting illness at least once a month. Among these cases, diarrhea accounted for 44%, gastrointestinal infections for 25%, cholera for 21%, typhoid for 5%, and other diseases for 5% [20].

The primary reason for the prevalence of waterborne infections in Pakistan is the contamination of drinking water with industrial waste and municipal sewage, combined with a lack of effective water disinfection methods and inadequate quality monitoring at treatment facilities. Hence, local authorities should monitor purification plants. Rusting pipelines should be renovated. Sufficient distance must be maintained between sewerage and water filtration plants to minimize the risk of contamination [21]. A systematic review by Fewtrell et al. estimated that improving water supply, sanitation, and handwashing could reduce diarrhea cases by 25%, 32%, and 45%, respectively. Additionally, household water treatment and safe water storage were found to lower diarrhea prevalence by 39% [22].

Beyond water contamination, a critical issue identified in this study was the lack of awareness and corrective measures taken by the community regarding safe drinking water practices. Despite 56.9% of respondents perceiving their drinking water as unsafe, corrective measures were not widely practiced. Only 15.7% of individuals reported taking precautions such as boiling water before drinking. This highlights a gap in public awareness and the need for proactive efforts to ensure water safety. Similarly, 71.6% of respondents recognized the need for a proper pipeline water supply and sewerage system; financial constraints and reliance on government intervention hindered progress. Notably, 79.4% of respondents were unwilling to pay for a pipeline water supply system, citing affordability issues (39.2%) and the belief that it was the government's responsibility (14.7%). Such attitudes underscore the necessity of government-led initiatives to improve rural water infrastructure and promote community-based interventions for water safety.

To mitigate the waterborne disease burden in rural areas, efforts should focus on increasing public awareness, promoting affordable water purification techniques, and ensuring proper maintenance of water sources. Regular monitoring of hand pump water quality, encouraging deeper borehole installations, and implementing low-cost household filtration systems could be effective strategies. In particular, subsidized household filters such as ceramic or biosand units offer a practical and low-maintenance solution for individual families. At the community level, NGO-led initiatives, such as shared borehole systems or small-scale purification plants, can provide safe drinking water while reducing the financial strain on households. Alongside these measures, corrective practices such as boiling water, solar disinfection, and safe storage should be encouraged through targeted awareness campaigns. Training local volunteers and health workers to monitor water quality and promote hygiene practices can strengthen community ownership and sustainability. An improved sanitation infrastructure combined with government-community collaboration, supported by NGOs and public-private partnerships, can help overcome financial barriers and foster long-term change. Collectively, these affordable, community-driven interventions, when integrated with broader policy-level initiatives, can play a crucial role in reducing contamination risks and lowering the prevalence of waterborne diseases in resource-limited rural settings.

Limitations

Similar to other studies, this study also has some limitations. Firstly, the cross-sectional design has restricted interpretation to associations rather than causation. Secondly, the relatively small sample size has limited the generalizability of the findings to wider populations. Thirdly, the reliance on self-reported data without clinical confirmation has introduced potential recall bias and misclassification of waterborne illnesses, particularly among respondents who were uncertain of their diagnosis. Fourthly, only descriptive analyses were performed, and the absence of inferential statistical testing limits the strength of evidence for the observed associations. Finally, the study was conducted in a single community, which may not fully represent the diversity of water access, sanitation infrastructure, and health outcomes in other settings across Pakistan. These limitations should be taken into account when interpreting the findings, and future studies with larger, more representative samples and robust statistical analyses are recommended.

## Conclusions

Waterborne diseases remain a significant public health concern in Pakistan, primarily driven by inadequate access to clean drinking water, poor sanitation, and industrial pollution. The findings of this study highlight the urgent need for improved water quality monitoring, infrastructure development, and targeted public health interventions to mitigate the burden of waterborne illnesses. To achieve this, specific policies such as subsidizing low-cost household filtration units, enforcing regular testing of hand pump and tube well water, and incentivizing the installation of deeper boreholes should be prioritized. Community-based programs, led by NGOs or in partnership with local governments, could establish small-scale purification plants, promote solar disinfection (SODIS), and provide hygiene education through local health workers. At the policy level, integrating rural water safety planning into existing health and sanitation frameworks would ensure sustainability. By adopting these actionable and affordable measures, significant reductions in waterborne disease prevalence can be achieved, ultimately improving health outcomes and resilience in vulnerable rural populations.
